# Supplementary material for: Single cell transcriptomics reveals the heterogeneity of the human cornea to identify novel markers of the limbus and stroma
Source: Sci Rep. 2021 Nov 5;11:21727. doi: 10.1038/s41598-021-01015-w (PMC8571304; doi:10.1038/s41598-021-01015-w)
Supplement: Supplementary file 1 — Supplementary Figures. [file 41598_2021_1015_MOESM1_ESM.docx]

**
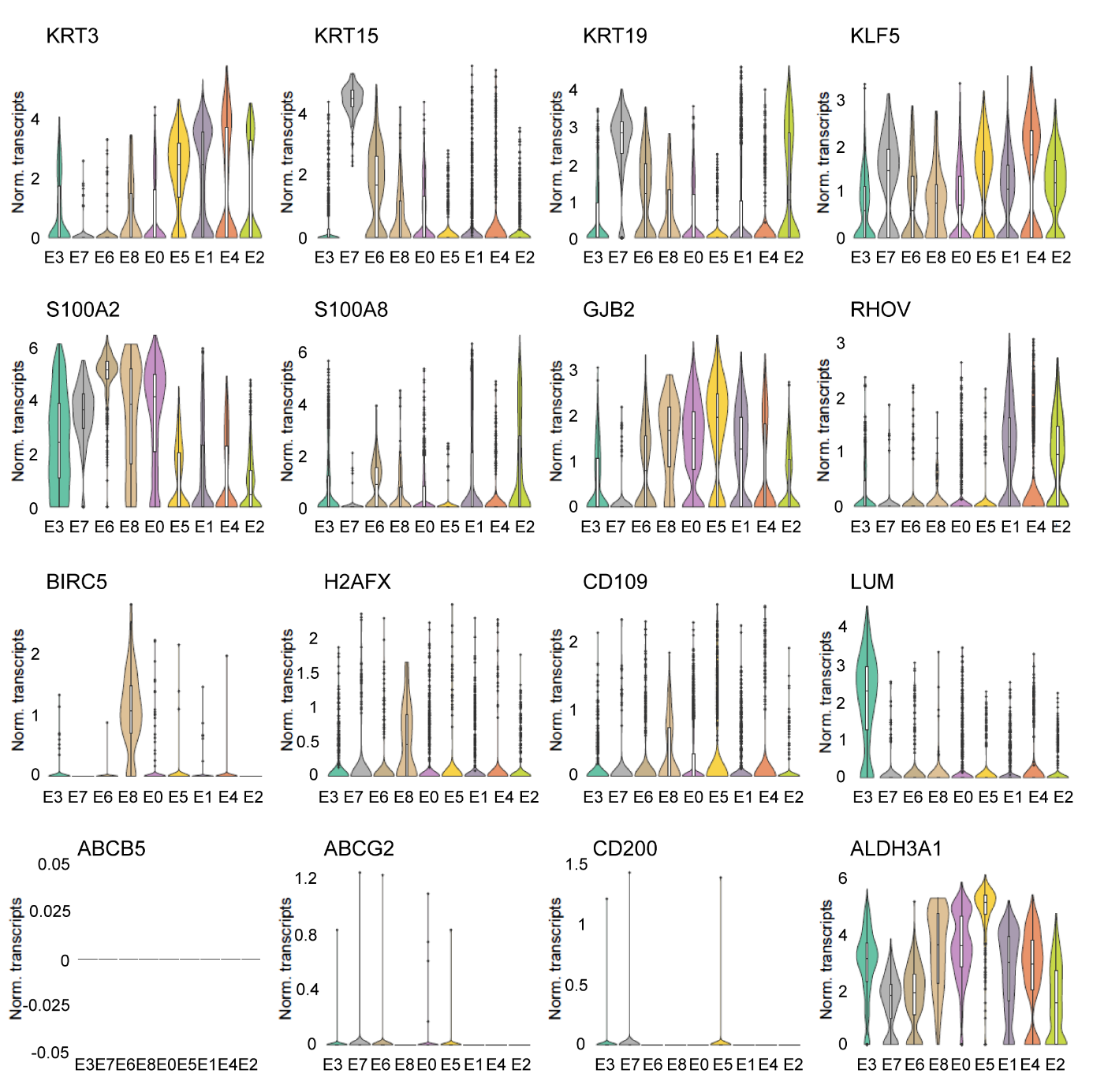
SUPPLEMENTARY FIGURE S1**

**Supplementary Figure S1.** Violin plots show additional marker genes for the identification of corneal epithelial clusters.

**SUPPLEMENTARY FIGURE S2**

**
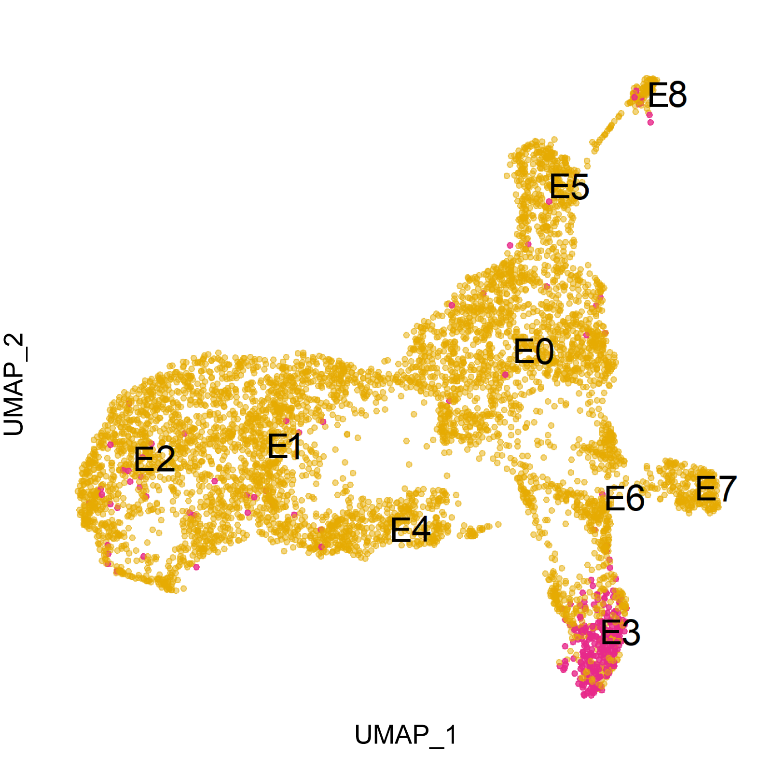
**

**Supplementary Figure S2.** Putative doublets were computationally identified using scDblFinder (v1.2.0) and epithelial cell cluster E3 was identified as a cluster of stromal-epithelial doublets.

**SUPPLEMENTARY FIGURE S3**


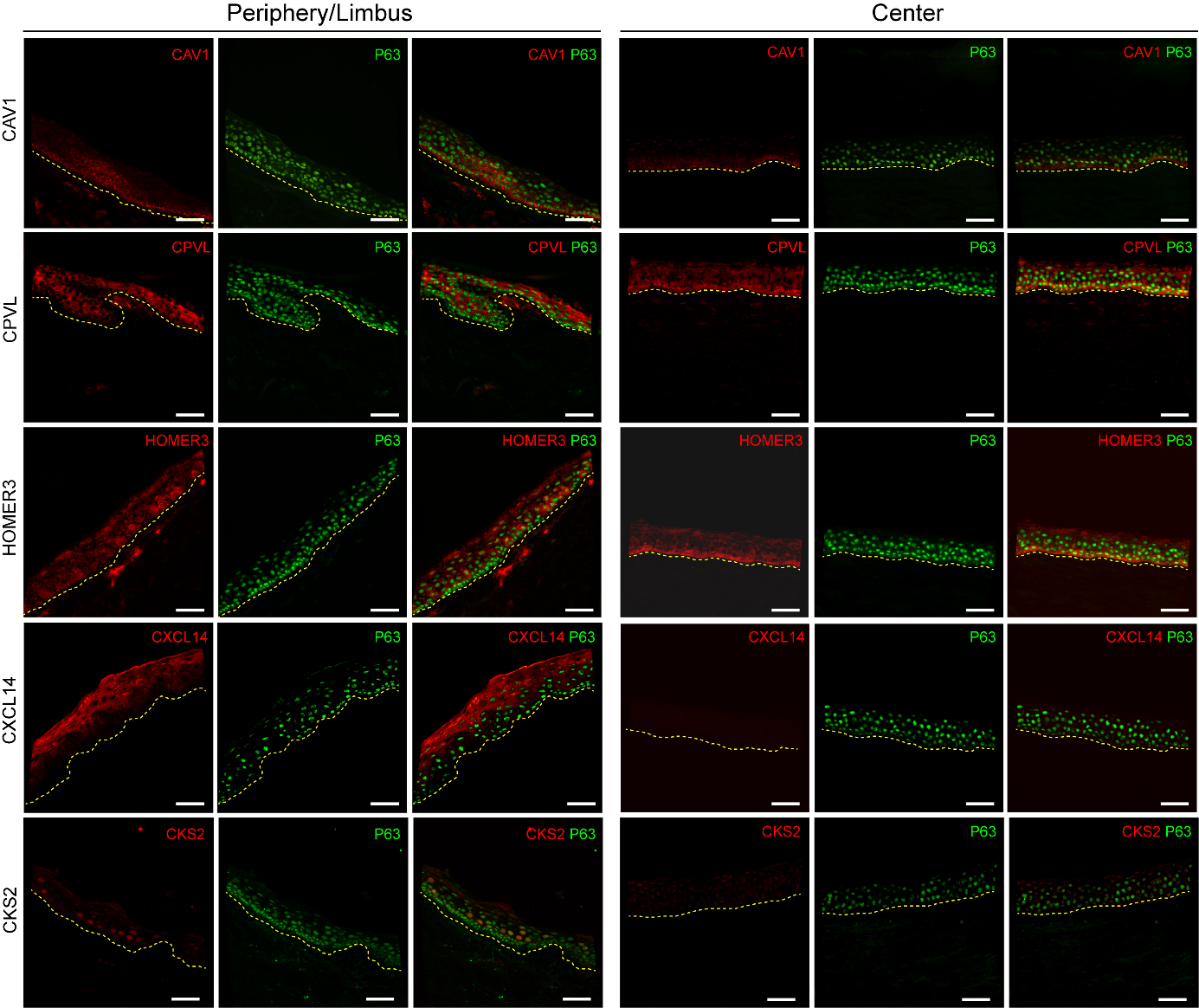


**Supplementary Figure S3.** Immunofluorescence of caveolin-1 (*CAV1*), CXCL14 and CKS2 (red) on human corneal tissue cryosections confirmed differential protein expression in the limbus/periphery, and absence (CKS2, and CXCL14) or minimal expression (caveolin-1) in the central cornea. Basal corneal epithelial cells retained minimal expression of both ΔNp63 and caveolin-1, suggesting their limbal origin. HOMER3 and CPVL were expressed in the limbus, but also in the central cornea, where central basal epithelial cells appeared to have higher expression. P63 (ΔNp63 or p63α) was used as a corneal epithelial limbal cell marker (green). The yellow dashed line indicates the boundary between epithelium (above) and stroma (below). Scale bars represent 50 μm.

**SUPPLEMENTARY FIGURE S4**


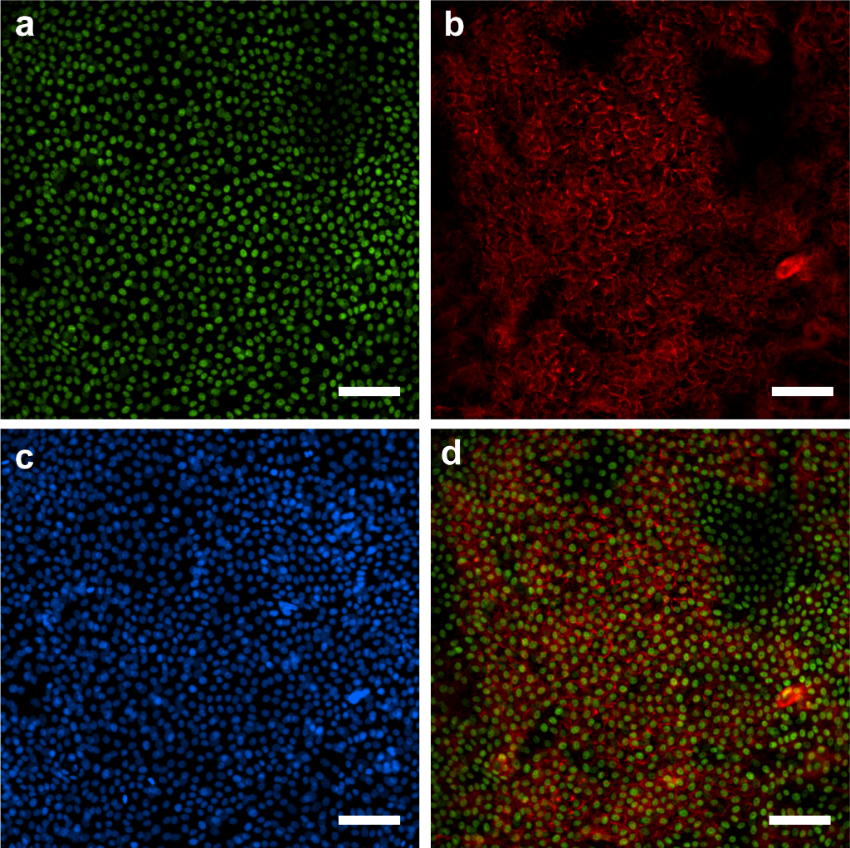


**Supplementary Figure S4.** Immunofluorescence analysis of caveolin-1 (*CAV1*) expression in primary cultured human corneal limbal epithelial stem cells. Limbal epithelial stem cells expressing ΔNp63 (a, green) also showed expression of caveolin-1 (b, red), as observed in the image overlay (d). Cell nuclei were stained with DAPI (c). These results suggest caveolin-1 could be a selective marker for corneal limbal stem cells. Scale bars represent 100 μm.

**SUPPLEMENTARY FIGURE S5**


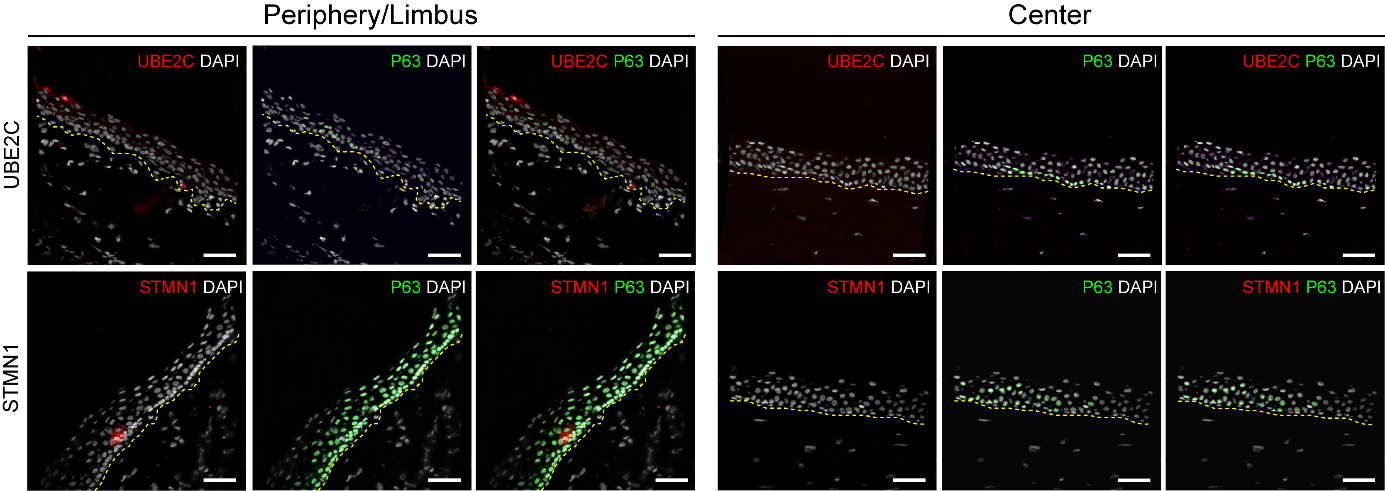


**Supplementary Figure S5.** Immunofluorescence of UBE2C and stathmin-1 (STMN1) on human corneal tissue cryosections confirmed differential protein expression in the limbus/periphery, and absence in the central cornea. P63 (ΔNp63 or p63α) was used as a corneal epithelial limbal cell marker (green). Cell nuclei were stained with DAPI (white). The yellow dashed line indicates the boundary between epithelium (above) and stroma (below). Scale bars represent 50 μm.

**SUPPLEMENTARY FIGURE S6**

**
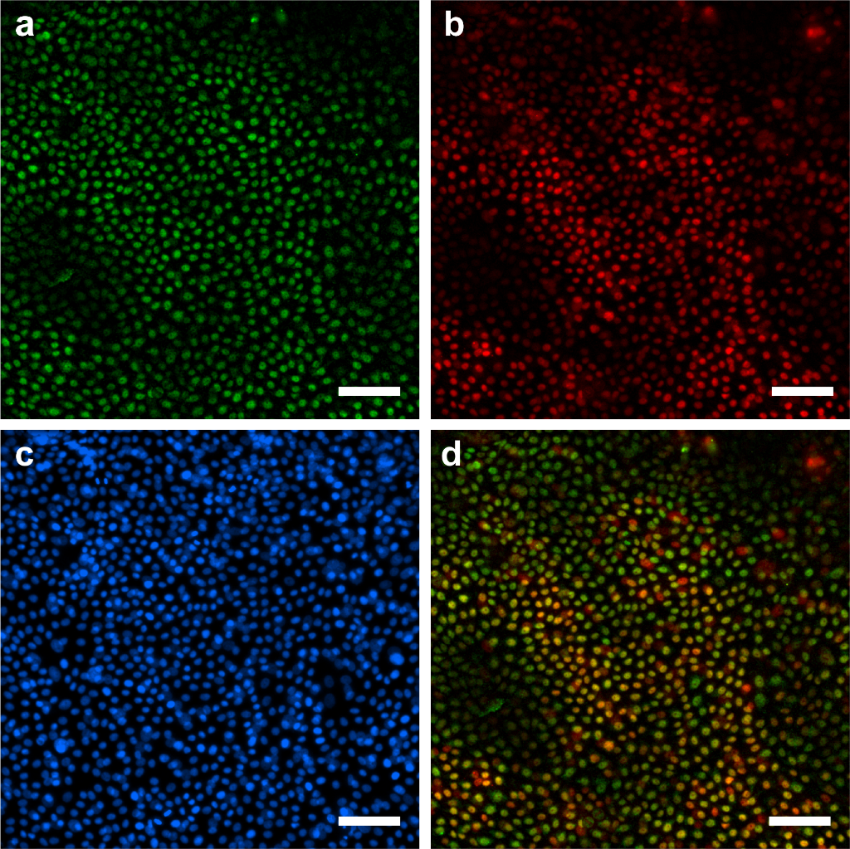
**

**Supplementary Figure S6.** Immunofluorescence analysis of cyclin-dependent kinase 2 (*CKS2*) expression in primary cultured human corneal limbal epithelial stem cells. Limbal epithelial stem cells expressing p63α (a, green) also showed expression of *CKS2* (b, red), as observed in the image overlay (d). Cell nuclei were stained with DAPI (c). Scale bars represent 100 μm.

**SUPPLEMENTARY FIGURE S7**

**
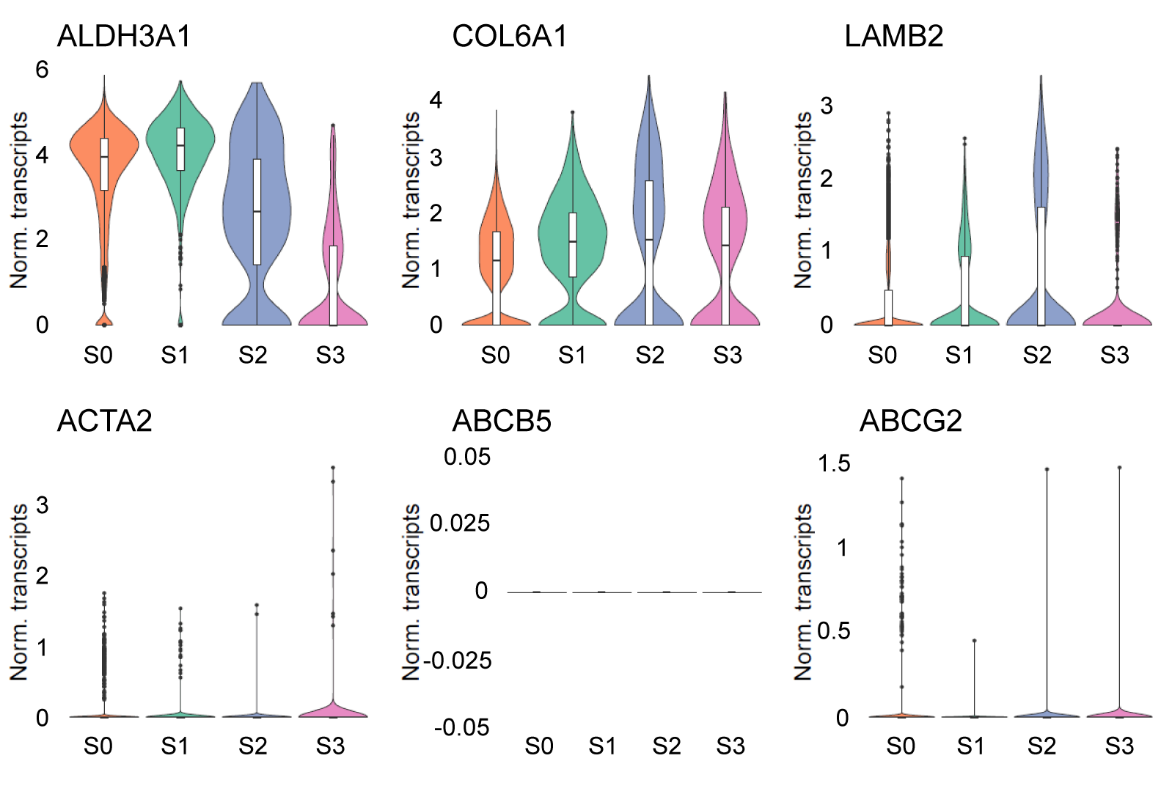
**

**Supplementary Figure S7.** Violin plots show additional marker genes for the identification of corneal stromal clusters.

**SUPPLEMENTARY FIGURE S8**

**
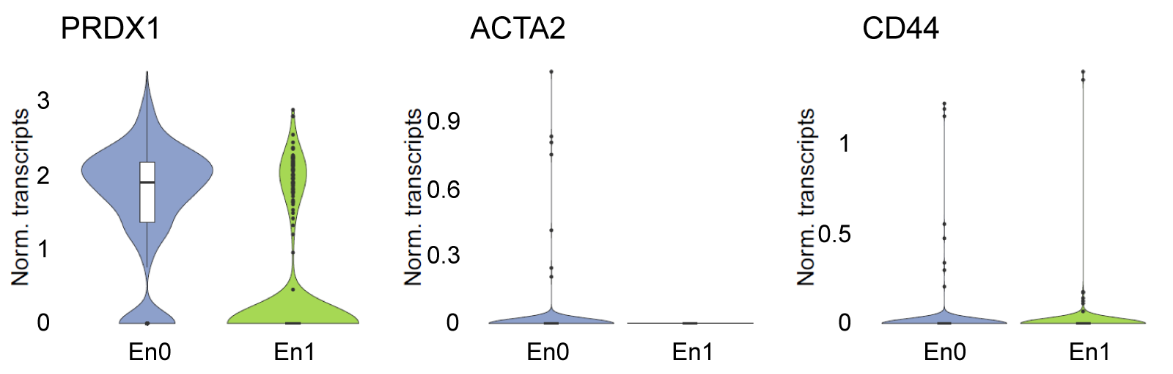
**

**Supplementary Figure S8.** Violin plots show additional marker genes for the identification of corneal endothelial clusters.

**SUPPLEMENTARY FIGURE S9**

In line with the study by Collin et al. 2021,^1^ our scRNAseq study shows differential expression of SARS-CoV-2 entry receptors *ACE2*, *TMPRSS2*, *TMPRSS4^2-4^* in the corneal epithelium, and *NRP1^5^* in both corneal epithelium and stroma. This results are in line with the hypothesis of the cornea as a potential SARS-CoV-2 entry site (Supplementary Figure S7). Finally, the lack of SARS-CoV-2 entry receptors in the corneal endothelium (Supplementary Figure S7), the most often selectively transplanted corneal layer, supports the safety of donor tissue for endothelial keratoplasty.


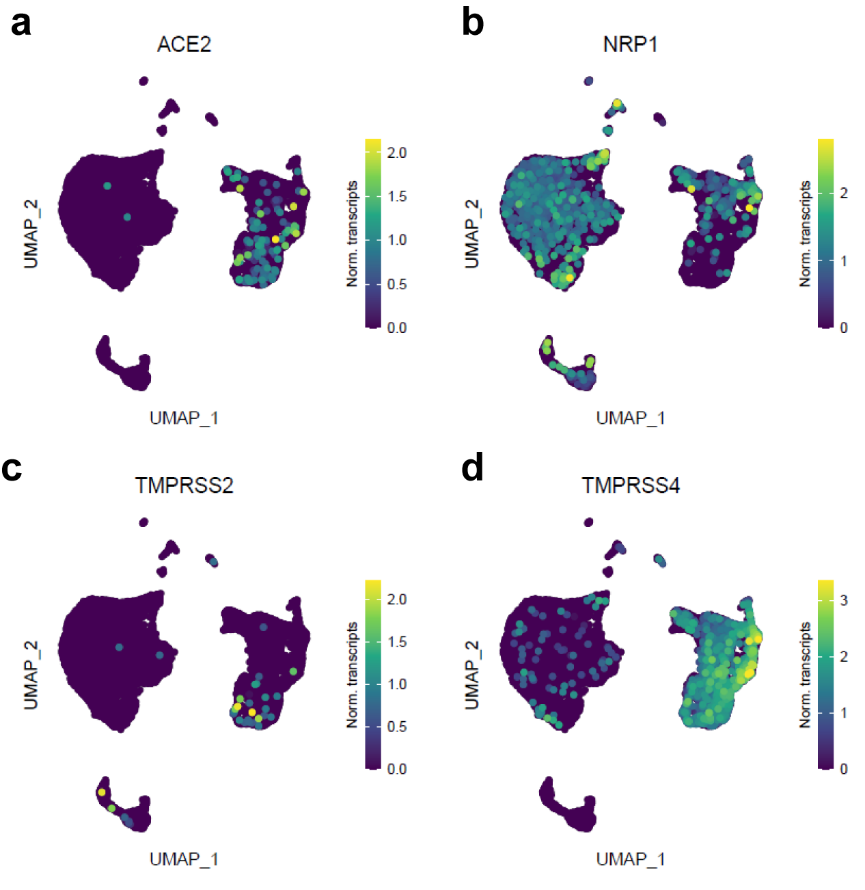


**Supplementary Figure S9.** Single cell transcriptome expression level UMAP of SARS-CoV-2 cell receptor *ACE2* (a) and receptor-associated *NRP1* (b), *TMPRSS2* (c), and *TMPRSS4* (d) protein expressing genes.

**REFERENCES**

1. Collin, J. *et al.* Co-expression of SARS-CoV-2 entry genes in the superficial adult human conjunctival, limbal and corneal epithelium suggests an additional route of entry via the ocular surface. *Ocul. Surf.* **19**, 190–200 (2021).

2. Yan, R. *et al.* Structural basis for the recognition of SARS-CoV-2 by full-length human ACE2. *Science.* **367**, 1444–1448 (2020).

3. Hoffmann, M. *et al.* SARS-CoV-2 Cell Entry Depends on ACE2 and TMPRSS2 and Is Blocked by a Clinically Proven Protease Inhibitor. *Cell* **181**, 271–280 (2020).

4. Zang, R. *et al.* TMPRSS2 and TMPRSS4 mediate SARS-CoV-2 infection of human small intestinal enterocytes. *Sci. Immunol.* **5**, eabc3582 (2020).

5. Cantuti-Castelvetri, L. *et al.* Neuropilin-1 facilitates SARS-CoV-2 cell entry and infectivity. *Science.* **370**, 856–860 (2020).
